# Consumer Acceptability of Dry Cured Meat from Cull Ewes Reared with Different Linseed Supplementation Levels and Feeding Durations

**DOI:** 10.3390/foods7060089

**Published:** 2018-06-11

**Authors:** Ana Guerrero, Carlos Sañudo, María del Mar Campo, Jose Luis Olleta, Erica Muela, Rosa M. G. Macedo, Francisco A. F. Macedo

**Affiliations:** 1 Department of Animal Production and Food Science, Instituto Agroalimentario (IA2), Universidad de Zaragoza—CITA, C/Miguel Servet, 177, 50013 Zaragoza, Spain; csanudo@unizar.es (C.S.); marimar@unizar.es (M.d.M.C.); olleta@unizar.es (J.L.O.); ericamola@hotmail.com (E.M.); rmgmacedo@uem.br (R.M.G.M.); fafmacedo@uem.br (F.A.F.M.); 2 Department of Animal Science, Universidade Federal, de Sergipe, Cidade Universitária, 49100-000 São Cristovão, Brazil

**Keywords:** cecina, ovine, sensory quality, traditional meat products

## Abstract

Dry cured meat—‘cecina’—is a traditional, although not well-known, dry product that could add value to cull ewes. Because of this, the aim of the study was to assess consumer acceptability of ‘cecina’ from cull ewes finished with different levels of linseed (5, 10 or 15%) for different periods before slaughtering (30, 50 or 70 days). One hundred and fifty consumers evaluated colour acceptability, fatness and odour, flavour and overall acceptability of ‘cecina’ from those 9 treatments. Additionally, habits of consumption of cured products and preferences for different species and willingness to pay for ‘cecina’ were investigated. Linseed supplementation was identified as the most important factor for sensorial attributes (*p* < 0.01), with the preferred ‘cecina’ being that with 5% and 10% supplementation. Feeding duration only modified the fatness acceptability (*p* < 0.01). ‘Cecina’ from small ruminants is a product consumed occasionally by the majority of participants; however, it presented an adequate overall acceptability. Consequently, elaborating ‘cecina’ would be a feasible strategy to improve the income of farmers.

## 1. Introduction

‘Cecina’ is a traditional meat product that can be elaborated from several species after salting, drying and, occasionally, smoking different pieces of meat, mainly back leg and sirloin [1]. This product is particularly appreciated and consumed in some countries of the Mediterranean area, but it has equivalents in many other areas of the world [2].

Depending on the region, species, joint and specific local variations on the production and fabrication processes, different products can be found with different denominations. Spanish ‘cecina’ resembles South African ’biltong’, South American ’charqui’, Italian ‘bresaola’ and Turkish ‘pastirma’ [1]. All of these have several common characteristics, such as their distinctive flavour (one of the key attributes for the consumer) and its local production following traditional processes.

In many Mediterranean countries, as currently happens in Spain, the ovine meat sector is important in the maintenance and sustainability of rural areas [3]. Usually, fresh meat from cull ewes has little economic value. However, dried and cured meat from those types of animals increases their commercial value [4], although dry-cured meat products from small ruminant are less frequent than from other species (beef, pork) [5]. Therefore, the transformation to ‘cecina’, which presumably presents a good overall acceptability according to previous studies [2], would have a positive economic outcome for breeders.

However, cull animals, which represent up to 40% of ewes in the flock [6], could need a slight improvement in their body score and conformation before slaughtering in order to improve their yield and carcass quality, and maybe the meat characteristics. With these aims, [7] studied the effect of the addition of different levels of linseed in the diet during different fattening periods on meat quality. Linseed was selected as an alternative ingredient to use in the finishing diets of those animals due to its energetic content, its nutritional profile, which is rich in *n*-3 polyunsaturated fatty acids, and its demonstrated ability to modify meat fatty acid composition, resulting in a healthier profile [8,9]. 

In addition, for obtaining market success of processed sheep meat, it is essential to know consumer sensory and hedonic perception [4]. Sensory attributes are one of the main characteristics underlying consumers’ overall liking, but other variables, such as familiarity with the product, socio-cognitive segmentation or genetic origins of the consumers would also be important topics to be considered and discussed [10] in order to better understand consumer acceptability of the tested product. 

Consequently, the current work examines the influence of the different levels of linseed supplementation and feeding duration in cull ewes on the consumer acceptability of the dry cured sheep meat ‘cecina’. 

## 2. Material and Methods 

### 2.1. Animals and Carcass Quality

Seventy-two ewes of the Rasa Aragonesa breed, a local medium wool breed, rustic type from the North East (Aragón) of Spain (for more information see [11]) were randomly selected from the cooperative Grupo Pastores^®^ from 5 commercial flocks, with all animals presenting similar characteristics of reproductive life, age (older than 6 years) and rearing conditions (semi-intensive systems). After their selection, cull ewes were transported to the facilities of the Veterinary Faculty of Zaragoza, where they were intensively finished based on concentrates and cereal straw. The experimental design was composed of 9 groups with 8 animals per group. The different experimental diets evaluated were isonitrogenous and isoenergetics.

A 3 × 3 factorial design was tested, which comprised 3 different percentages of *Linum usitatissimum* (linseed), which was supplied as whole seeds on the concentrate diet (linseed supplementation level—LSL): 5%, 10% or 15%. Also, 3 different finishing duration periods (FD) where evaluated: 30, 50 or 70 days.

Productive parameters for growing and animal performance, such as live weight, body condition score, average daily gain, were compiled throughout the experiment, and were compiled in [7]. When the corresponding finishing periods were reached, animals were transported (<3 h) to an EU-licensed abattoir to be slaughtered after a resting period of 15–20 h. Then, carcasses were kept at between 2 and 4 °C overnight. Twenty-four hours post-slaughter, the left side of the carcass was transported refrigerated to the Meat Quality Laboratory of the Veterinary Faculty of Zaragoza, where the hind limb was excised and used to elaborate the ‘cecina’ to be evaluated by consumers.

### 2.2. Curing Procedure

Each hind limb, after being excised, was vacuum packed and sent with 4 days of ageing to the food industry, where they were processed (Secadero Sierra Maestrazgo®, Castellote, Teruel, Spain) in order to obtain ‘cecina’ (cure meat) with the same methodology described in [2].

The curing procedure consisted of piling and covering the surface of the hind legs with marine salt in a cold room, inverting the location of the pieces periodically to guarantee the homogenous penetration of a convenient amount of salt on the pieces. Later, salt residues on the surface of the meat pieces were eliminated by washing them with cold water, and for salt equilibration, pieces were hung in a cold room (5 °C; 80% humidity) for a period of between 45 and 50 days. Finally, drying was conducted by hanging the meat pieces in natural conditions over winter (2 months, room temperature of 15 °C, relative humidity of 85–90%). During the drying process, biochemical reactions occur, giving the characteristic and typical flavours and textures associated with ‘cecina’ by ripening [12]. Once the technician had certified that each ‘cecina’ batch had finished its curing process and it was ready for consumption, the legs were retired, deboned, vacuum packed, and kept at 4 °C until consumer analysis.

### 2.3. Consumer Test

Home-based tests [13] were used to determinate the acceptability of ‘cecina’. In the trial, 150 local consumers were selected by gender and age according to the Spanish national profile [14] were involved. 54% of participants were women and 46% men. With regard to the age interval, 22.7% of consumers were between 18 and 34 years old; 47.3% were between 35 and 50 years old, and 30.0% were between 51 and 80 years old. All participants were of Spanish origin and were residents in Zaragoza. Consumers came from 46 different families, with 1 to 4 members each one.

Participants filled in a survey including closed questions with multiple choices, in order to obtain the frequency of consumption of cured products, knowledge about species from which it is possible to obtain ‘cecina’, and willingness to pay for those products.

The deboned ‘cecina’ was sliced with a continuous blade machine (Braher® mod. DSB28, Braher International, San Sebastian, Spain) into 1.5-mm-thick slices and packaged in three-digit-coded aluminium foil and vacuum packed together with all of the samples that each consumer was to evaluate.

Each consumer assessed 9 samples, one from each dietary treatment studied (3 linseed supplementation level (LSL) × 3 finishing duration periods (FD)). Consumers were asked to eat unsalted toasted bread and rinse their mouth with water before evaluating each sample. For each sample, consumers evaluated the acceptability of the following attributes: colour, fatness, odour, flavour and overall, by using a hedonic 9-point scale ranging from 1 (dislike extremely) to 9 (like extremely). The neutral central point (neither like nor dislike) had been deleted in an attempt to force the consumer to make a decision in a positive or negative way [15].

### 2.4. Statistical Analysis

Analysis of variance was performed using the General Linear Model (GLM) procedure of the SPSS statistical package (v.19.0, IBM Corp., Armonk, NY, USA). For consumer preference, linseed supplementation level (LSL), feeding duration (FD) and their interactions were considered as fixed effects, and consumer as a random effect.

The model was as follows:

*Y_ijk_* = *µ* + *LSL_i_* + *FD_j_* + *A_k_* + (*LSL_i_* × *FD_j_*) + *e_ijk_*

where *Y_ijk_* is the dependent variable; *µ* is the population average; *LSL_i_* is the fixed effect of linseed supplementation level (5, 10 or 15%); *FD_j_* is the fixed effect of supplementation period (30, 50 or 70 days); *A_k_* is the random effect of the consumer; (*LSL_i_ × FD_j_*) is the interaction effect of linseed supplementation and period; and *e_ijk_* is the random error. The mean and standard error of the mean (SEM) were calculated. Differences between means were evaluated using Duncan’s Multiple Range Test, (*p ≤* 0.05). A hierarchical cluster analysis was performed on overall acceptability using XLSTAT 16.5 in order to identify similarities among consumers.

## 3. Results and Discussion

### 3.1. Characterisation of Consumer Sample 

The frequencies of consumption of different meat products by the participants of the study are compiled in Table 1. It can be observed that ‘cecina’ is consumed very sporadically by the majority of participants (82.8%), presenting lower frequencies of monthly and weekly consumption (8.3% and 1.4%, respectively). However, consumption of other dry cured products derived from pork, especially ham, is higher. Those results agree with other studies [16], in which 26% of participants consumed ham daily, and 67% several days per week. In Spain, there is a high culture of ham consumption (2.41 kg per capita, which represents about a quarter of the total meat products consumed [17], and a broad knowledge of its characteristics and brands [16]. However, in other European countries consumption of ham is less than once per week (Belgium, Denmark, Poland, Greece or Germany) [18], or very occasional in some other countries with high meat consumption such as Brazil [19].

Related to willingness to pay for ‘cecina’ (Table 2), participants were informed that the average price of cured ham in the market was 15 €/kg, and different possible prices for ‘cecina’ were subsequently presented (ranging from 10.5 €/kg to 19.5 €/kg). 15.2% of participants would pay the same price for ‘cecina’ as for ham (15 €/kg), 27.6% would pay a higher price for the product ‘cecina’ (16.5–18 €/kg), with a low percentage (2.1%) being willing to pay up to 19.5 €/kg. Approximately half of the answers indicated that consumers would pay a price lower for ‘cecina’ than the price of ham. Consumer education into “gourmet gastronomy” would be an alternative to improve success in the promotion and choice of a not-well-known product [19], such as ‘cecina’.

Another question in the survey explored the knowledge related to the varieties of ‘cecina’ available. Consumers were asked to mark the species that they thought had ‘cecina’ in the market, with the possibility of marking multiple answers. The most popular species for ‘cecina’ that the majority of consumers knew were: deer (81.4%), cow (71.7%), wild pig (69.0%) and horse (62.8%). ‘Cecina’ from small ruminants was less popular; only 37.2% of consumers thought that ‘cecina’ from goat existed, and 31.0% of consumers thought the same for sheep. ‘Cecina’ from pig, duck or rabbit were little known by consumers (37.2%, 22.1% and 4.8%, respectively). In this sense, in Spain, the only ‘cecina’ with Protected Geographic Indication (PGI) is from beef (‘Cecina de León’).

These results can help to understand the answers compiled in Table 3 about the species that consumers preferred for ‘cecina’, because consumers usually prefer already known and experienced products, and in Spain the most popular ‘cecina’ product comes from beef. In any case, there is a small niche of consumers (11.7%) who chose ‘cecina’ from sheep as one of the three preferred first-choice options. Data from several sensory studies [10,20] has shown that familiar foods, and exposure to new foods, contribute to reducing the possible initial neophobia towards novel or unfamiliar foods.

### 3.2. Consumer Acceptability

The addition of different levels of linseed or the different duration of the feeding period did not modify (*p* > 0.05) the colour acceptability of ‘cecina’ (Table 4). This attribute was scored between 6.41–6.58 on a 9-point scale, which would be equivalent to answers like slightly to moderate. In the current data, no variation in colour would be expected, because the process of elaboration was identical for all treatments. Even the modification of some procedures, such as tumbling treatments [5] reported no significant differences in instrumental colour variables such as lightness, redness or yellowness. Consequently, it was expected that consumers did not report differences in colour acceptability. Differences on ‘cecina’ colour, such as those reported by [12] in beef, were associated with the different durations of drying.

Acceptability of fat quantity on ‘cecina’ samples from 15% of supplementation presented lower values than 5% or 10% (*p* < 0.001), which correspond to a Body Condition Score (BCS) of 3.33, probably considered the quantity of fat excessive. However, with regard to feeding duration (*p* < 0.01), samples from 30 days presented lower values than those from 50 days, having longer fattening periods (70 days) and intermediate scores. Products from the 30 days (BCS of 2.42) were probably considered as too lean.

On the sensorial attributes evaluated (odour, flavour and overall acceptability) linseed level was a significant factor (*p* < 0.01), with the 15% level presenting the lowest acceptabilities, but feeding duration did not modify (*p* > 0.05) those variables. However, there were significant interactions (*p* < 0.01) between both factors (linseed level and feeding duration).

Odour is an important attribute in dry cured products. As [5] reported, the most numerous compounds in the dry-cured lamb legs are straight-chain aldehydes followed by alcohols, ketones and benzene compounds. These compounds have been described in volatile profiles of dry-cured ruminant or pork meat products [1,21]. Variations in the ripening time and tumbling modified the presence of several compounds, such as 3-methylbutanal from leucine and phenylacetaldehyde from phenylanine. These compounds are considered to be major contributors of dry-cured ham flavour. The highest value for odour acceptability was associated with ‘cecina’ with 10% linseed supplementation and 30 days of fattening, with 6.29 points on a 9-point scale. This value did not differ statistically from those scored at any feeding period of the lowest level of supplementation 5%, or from those with 70 days of fattening but with 10% or 15% supplementation. The lowest acceptabilities were reported for 15% with 30 or 50 days of fattening.

In dry-cured products such as ham, taste is considered to be a key attribute influencing consumer overall liking, and colour, flavour and adequate saltiness are considered basic quality signals by consumers [4]. In the current study, differences in colour or saltiness did not exist, due to the identical processing. Flavour and overall acceptability presented the same consumer evaluation behaviour, and in meat it is also common to find a high correlation between the sensory attribute flavour and the overall acceptability of the evaluated product [22], which explains the equivalent behaviour of both variables. The preferred combination of treatments that present the highest punctuation (6.11 and 6.16, respectively, for flavour and overall acceptability) was ‘cecina’ from the lowest linseed supplementation and the lowest feeding duration (5% and 30 days). However, those values did not statistically differ from those of any of the combinations of feeding duration with 5 or 10% linseed, or 15% at 70 days. The lowest score came from ‘cecina’ with 15% and 30 days of fattening.

Volatile Free Fatty Acids (FFA) are formed by lipolysis, and FFA alkyl esters seem to be formed by microbial esterification during ripening [1,5]. Given that those compounds are associated with ripened flavour in cured meat products and increase through the ripening [5], they contribute directly to taste and indirectly to odour and flavour by means of aroma compounds [12].

Differences in odour and flavour associated with linseed level are probably linked to the modifications in the fatty acid profile produced by linseed supplementation [7], occurring as an increment in the percentage of total PUFA and *n*-3 PUFA. The levels of lipid oxidation-derived compounds are relevant for the flavour of dry-cured meat products [5].

In a general way, it can be observed that the overall acceptability of ‘cecina’ presented similar values (slighter lower) than those reported by [2] for Spanish consumers (6.00–6.83 points). Therefore, ‘cecina’ from cull ewes would presumably present an adequate acceptability and good commercial reception in Spain. 

According to data from the current and the previous study [2], ‘cecina’ with lean or medium fat levels is preferred by consumers, so it is not necessary to have long feeding durations for finishing cull ewes (30 days would be enough). Also, low levels of linseed (5%) would be enough if the final product destination were the elaboration of ‘cecina’, or even up to 10%, if the aim were the consumption of fresh meat [7]. 

### 3.3. Cluster Analyses

Consumers present different preferences, which leads to the existence of several market niches. Three clusters (groups) of consumers with different preferences related to the overall acceptability of ‘cecina’ were identified (Table 5).

For the first cluster identified, only linseed level had an influence (*p* < 0.001) on the overall acceptability of ‘cecina’. This cluster represented 14.7% of consumers in the study, and the main characteristics of this group were the rejection of the product, presenting low acceptability scores ranging between (2.47–3.30 points), especially ‘cecina’ from the highest level of linseed supplementation (15%), which presented the lowest score. In this group of people, the percentage of male consumers was 45.5% and the age of participants was almost equivalent for each of the 3 age groups, with consumers younger than 30 being slightly more abundant, 36.4% vs. 31.8% for the other two groups. Related to answers of the questionnaire, in this group, 90.9% of consumers eat ‘cecina’ very sporadically. Also, 90.9% of the group would pay less than 15 €/kg (which was the prize of reference for ham) for ‘cecina’, and none of the participants would also have preferred the ‘cecina’ of sheep in one of the top 3 species preferences, although only 13.6% thought that this type of ‘cecina’ existed in the market. 

Cluster 2 included the most participants (58.0% of the sample). For this group the linseed level was a significant factor (*p* < 0.05) in acceptability, with 10% supplementation being preferred with respect to the other supplementation levels. However, although feeding duration did not present statistical differences, there was a significant interaction between both factors (*p* < 0.001). The overall acceptability score for all treatments in this group was good (from 6.71 to 7.22), higher than the average overall score. The highest scores were for the 10% supplementation level group with 30 or 70 days of fattening, and 15% with 50 days of fattening. The worst score treatment was for the 5% supplementation group with 50 days of fattening; but even then, that score was 6.71 points. With regard to socio-demographic characteristics, the cluster was formed of 47.1% men, with the most abundant age range being between 31 and 50 years (43.7%), and the least predominant consumers being those younger than 30 years (20.7%). According to the survey, 20.7% of this group consumed ‘cecina’ at least one at month, which showed in that this cluster was more familiar with the product than the other clusters, and explained the higher scores for ‘cecina’ than cluster 1. 3.53.6% of the cluster would pay a price equivalent to those given as reference for ham (15 €/kg: 20.7% of them) for ‘cecina’, or higher, up to 19.5 €/kg. 39% of these consumers think that ‘cecina’ from sheep exists on the market and 16.6% of answers indicate that this ‘cecina’ would be one of the top 3 preferred.

The last group identified included 27.3% of participants. Similar to cluster 2, linseed level was a significant factor (*p* < 0.05); however, the least preferred level of supplementation was 15%, with the acceptabilities for supplementation at 5 or 10% being similar. Also, there was a significant interaction between linseed supplementation level and fattening duration (*p* < 0.001). ‘Cecina’ from treatment with 15% linseed for 30 or 50 days, as well as, ‘cecina’ from 10% and 70 days of finishing, had the lowest acceptabilities of the different clusters, with scores lower than 5 points. Averages for acceptability in this cluster varied from 4.20 to 6.00 points. Cluster 3 was composed of a minority of men (43.9%). Consumers between 31–50 years old accounted for 63.4%, and older than 51 represented 17.1% of the cluster, and 19.5% were younger than 30 years. With regard to the frequency of ‘cecina’ consumption, in spite of the majority of people having ‘cecina’ very sporadically (85.4%), there were 4.9% that consumed the product once a month. 34.2% of consumers would pay more than 15 €/kg for ‘cecina’ and 53.6% would pay less than the price of reference (<15 €/kg). 7.4% of participants marked ‘cecina’ from sheep as one of the three preferred species of ‘cecina’ to consume.

## 4. Conclusions

According to the findings from the current study, ‘cecina’ from cull ewes presented an adequate acceptability for local consumers. The duration of the finishing period scarcely affected ‘cecina’ attributes and its acceptability, with the effect of linseed supplementation level being more significant. Low levels of fat are usually preferred by consumers, therefore short finishing periods (30 days) are enough to improve and homogenize the carcasses of cull ewes, and supplementations with low levels of linseed (5% or 10%) would be recommended based on ‘cecina’ attributes and economical profitability. This product would increase the income from adult animals, which usually have a low market value. Consequently, production of ‘cecina’ would be a feasible strategy to improve the yields of animals and income of farmers, having a positive economic outcome for breeders.

## Figures and Tables

**Table 1:** Frequency of consumption of different meat products (percentage; *n* = 145 consumers).

	Almost Every Day	2–3 t/w	Once/w	Once/15 d	Once/m	Ocassionally	Not Answer
Cecina	0.0	1.4	1.4	1.4	8.3	82.8	4.8
Ham	10.3	50.3	24.1	9.0	4.8	1.4	0.0
Cured loin	2.1	9.0	26.2	18.6	21.4	19.3	3.4
Chorizo	3.4	20.0	28.3	17.9	12.4	17.2	0.7
Fuet	2.1	16.6	20.0	13.1	15.9	26.9	5.5
Others	6.2	4.8	11.7	6.9	6.2	22.8	41.4

t: times; w: week; d: day; m: month.

**Table 2:** Price ranges that consumers would be willing to pay for ‘cecina’ (percentage; *n* = 145 consumers).

Price Ranges	Percentage of Consumers
10.5 €/kg	24.1
12.0 €/kg	17.2
13.5 €/kg	10.3
15.0 €/kg	15.2
16.5 €/kg	11.7
18.0 €/kg	15.9
19.5 €/kg	2.1
Not answer	3.4

**Table 3:** Preference of ‘cecina’ from different species (percentage; *n* = 145 consumers).

	1st Preferred	2nd Preferred	3rd Preferred
Horse	9.8	10.4	19.5
Cow	29.4	12.6	14.3
Sheep	2.8	4.4	4.5
Goat	4.9	4.4	6.8
Rabbit	-	0.7	1.5
Deer	26.6	31.9	23.3
Wild pig	12.6	28.1	15.0
Pig	8.4	5.2	12.0
Duck	5.6	2.2	3.0

**Table 4:** Acceptability of sensory attributes of ‘cecina’ from cull ewes fed with different linseed levels and fattening periods (*n* = 150 consumers).

		LSL			FD			5% LSL			10% LSL			15% LSL					
	5%	10%	15%	30 ^d^	50 ^d^	70 ^d^	30 ^d^	50 ^d^	70 ^d^	30 ^d^	50 ^d^	70 ^d^	30 ^d^	50 ^d^	70 ^d^	SEM	LSL	FD	LSL × FD
Colour	6.41	6.58	6.46	6.48	6.46	6.51	-	-	-	-	-	-	-	-	-	0.045	0.109	0.859	0.475
Fatness	6.58 ^b^	6.65 ^b^	6.36 ^a^	6.44 ^a^	6.65 ^b^	6.50 ^a,b^	-	-	-	-	-	-	-	-	-	0.043	0.001	0.034	0.307
Odour	-	-	-	-	-	-	6.06 ^bc^	6.20 ^b,c^	6.08 ^bc^	6.29 ^c^	5.86 ^a,b^	5.95 ^a,b,c^	5.66 ^a^	5.66 ^a^	6.04 ^b,c^	0.053	0.001	0.384	0.004
Flavour	-	-	-	-	-	-	6.11 ^c^	5.75 ^b,c^	5.83 ^bc^	5.95 ^b,c^	5.90 ^b,c^	5.85 ^b,c^	5.29 ^a^	5.63 ^b^	5.90 ^b,c^	0.059	0.003	0.580	0.002
Overall acceptability	-	-	-	-	-	-	6.16 ^c^	5.88 ^b,c^	5.94 ^bc^	6.11 ^bc^	5.97 ^b,c^	5.84 ^b,c^	5.39 ^a^	5.76 ^b^	6.03 ^b,c^	0.056	0.004	0.736	0.000

^a–c^, Lowercase letters indicate statistical differences in the same row between treatments (*p* ≤ 0.05). SEM: standard error of mean; LSL: effect of linseed supplementation level (*p*-value); FD: effect of feeding duration (*p*-value); LSL × FD: interaction between linseed supplementation level and feeding duration (*p*-value). Based on a 9-point scale: (1: dislike extremely; 9: like extremely).

**Table 5:** Overall acceptability scores of ‘cecina’ from cull ewes fed with different linseed levels and fattening periods in three clusters of consumers (*n* = 150).

			LSL			FD			5% LSL			10% LSL			15% LSL					
	%	5%	10%	15%	30 ^d^	50 ^d^	70 ^d^	30 ^d^	50 ^d^	70 ^d^	30 ^d^	50 ^d^	70 ^d^	30 ^d^	50 ^d^	70 ^d^	SEM	LSL	FD	LSL × FD
Cluster 1	14.7	3.30 ^b^	2.94 ^b^	2.47 ^a^	2.98	2.83	2.89	-	-	-	-	-	-	-	-	-	0.095	0.000	0.725	0.661
Cluster 2	58.0				-	-	-	7.08 ^b,c^	6.71 ^a^	6.97 ^a,b,c^	7.20 ^c^	7.02 ^a,b,c^	7.22 ^c^	6.74 ^a,b^	7.16 ^c^	6.92 ^a,b,c^	0.042	0.029	0.763	0.010
Cluster 3	27.3				-	-	-	5.66 ^c^	5.54 ^c^	5.22 ^b,c^	5.37 ^c^	5.46 ^c^	4.51 ^a,b^	4.20 ^a^	4.54 ^ab^	6.00 ^c^	0.098	0.046	0.752	0.00

^a–c^, Lowercase letters indicate statistical differences in the same row between treatments (*p* ≤ 0.05). SEM.: standard error of mean; LSL: effect of linseed supplementation level (*p*-value); FD: effect of feeding duration (*p*-value); LSL × FD: interaction between linseed supplementation level and feeding duration (*p*-value). Based on a 9-point scale: (1: dislike extremely; 9: like extremely).
